# Differential Reorganization of SMA Subregions After Stroke: A Subregional Level Resting-State Functional Connectivity Study

**DOI:** 10.3389/fnhum.2019.00468

**Published:** 2020-02-28

**Authors:** Huaigui Liu, Wangli Cai, Lixue Xu, Wei Li, Wen Qin

**Affiliations:** ^1^Department of Radiology, Tianjin Medical University General Hospital, Tianjin, China; ^2^Department of Radiology, Shanghai Tenth People’s Hospital, Tongji University School of Medicine, Shanghai, China; ^3^Department of Radiology, Beijing Friendship Hospital, Capital Medical University, Beijing, China; ^4^Department of Radiology, Tianjin Medical University Cancer Institute & Hospital, Tianjin, China

**Keywords:** stroke, supplementary motor area, parcellation, resting-state fMRI, reorganization

## Abstract

**Background and Purpose**: The human supplementary motor area (SMA) contains two functional subregions of the SMA proper and preSMA; however, the reorganization patterns of the two SMA subregions after stroke remain uncertain. Meanwhile, a focal subcortical lesion may affect the overall functional reorganization of brain networks. We sought to identify the differential reorganization of the SMA subregions after subcortical stroke using the resting-state functional connectivity (rsFC) analysis.

**Methods**: Resting-state functional MRI was conducted in 25 patients with chronic capsular stroke exhibiting well-recovered global motor function (Fugl–Meyer score >90). The SMA proper and preSMA were identified by the rsFC-based parcellation, and the rsFCs of each SMA subregion were compared between stroke patients and healthy controls.

**Results**: Despite common rsFC with the fronto-insular cortex (FIC), the SMA proper and preSMA were mainly correlated with the sensorimotor areas and cognitive-related regions, respectively. In stroke patients, the SMA proper and preSMA exhibited completely different functional reorganization patterns: the former showed increased rsFCs with the primary sensorimotor area and caudal cingulate motor area (CMA) of the motor execution network, whereas the latter showed increased rsFC with the rostral CMA of the motor control network. Both of the two SMA subregions showed decreased rsFC with the FIC in stroke patients; the preSMA additionally showed decreased rsFC with the prefrontal cortex (PFC).

**Conclusion**: Although both SMA subregions exhibit functional disconnection with the cognitive-related areas, the SMA proper is implicated in the functional reorganization within the motor execution network, whereas the preSMA is involved in the functional reorganization within the motor control network in stroke patients.

## Introduction

The human supplementary motor area (SMA) consists of two functionally dissociated subregions (Klein et al., 2007; Nachev et al., 2008; Kim et al., 2010; Zhang et al., 2011). The caudal SMA proper is anatomically connected with brain regions of the motor execution network, including the primary motor cortex (M1), spinal cord, basal ganglia, and cerebellum, and involved in motor execution (Muakkassa and Strick, 1979; Dum and Strick, 1991, 2005; Galea and Darian-Smith, 1994; He et al., 1995; Maier et al., 2002). The rostral preSMA mainly connects with the prefrontal cortex (PFC; Luppino et al., 1993; Lu et al., 1994; Wang et al., 2005) and involves higher-level processing, such as motor control and attention (Nakata et al., 2008; Boehler et al., 2010; Krüger et al., 2013; Cummine et al., 2017; Obeso et al., 2017). The resting-state functional connectivity (rsFC) patterns of the SMA subregions have been investigated in healthy subjects (Zhang et al., 2011). Similar to the anatomical connection patterns, the SMA proper and preSMA, respectively, showed rsFC with the sensorimotor and prefrontal areas, although both subregions were connected to the insula.

The subcortical infarction frequently impairs the internal capsule, corona radiata, and basal ganglia, which have direct or indirect anatomical fibers with other cortical regions (Alexander et al., 1986; Dum and Strick, 1991). The lesions interrupt the integrity of fibers that pass through them and subsequently affect the directly or indirectly connected distal cortical areas (Grefkes and Fink, 2014). Previous studies have explored that the focal subcortical lesions can trigger remote effects on the function of brain networks after stroke (Dancause, 2006; Grefkes et al., 2008; Dubovik et al., 2012; Grefkes and Fink, 2014), which may account for various behavioral deficits, such as motor deficit (Wang et al., 2010), aphasia (de Boissezon et al., 2005; Choi et al., 2007), spatial neglect (He et al., 2007), cognitive impairment (Stebbins et al., 2008; Gottesman and Hillis, 2010), and so on. Motor deficit is the most common symptom in stroke, and motor recovery has been associated with functional reorganization of the motor network (Duncan et al., 2000). All previous studies on the reorganization of the SMA after stroke have treated the SMA as a whole and reported increased activation in the SMA (Tombari et al., 2004; Jaillard et al., 2005; Tang et al., 2015; Chen et al., 2018) and increased effective connectivity of the SMA in chronic stroke patients when they perform motor or motor imagery tasks (Mintzopoulos et al., 2009; Sharma et al., 2009; Rehme et al., 2011). However, the rsFC alterations of the SMA after stroke have never been studied at the level of the subregion. Considering the different connectivity patterns and functions of the SMA subregions and the importance of the SMA in motor recovery after stroke, we hypothesized that the rsFCs of the SMA subregions may show different reorganization patterns in stroke patients.

Recently, the SMA has been consistently parcellated into the SMA proper and preSMA based on different MRI modalities (Johansen-Berg et al., 2004; Kim et al., 2010; Zhang et al., 2011). This parcellation framework is generally carried out using unsupervised clustering methods according to the similarity in either anatomical or functional connectivity profiles between voxels. In contrast to the traditional parcellation methods that are mainly based on local information (such as gyrification landmark, cytoarchitectonics, and activations, etc.), the connectivity-based clustering methods can parcellate the brain areas into several subregions that have similar connectivity profiles within each subregion, while much different across them. Thus, this strategy is more preferable for a connectivity-related study in contrast to traditional subregions based on local information (Fan et al., 2016). These methods have been applied to human and animal *in vivo* brain studies and demonstrate high reliability and accuracy (Wang et al., 2015, 2019; Schaefer et al., 2018). In the present study, we automatically parcellated the SMA based on the functional connectivity profiles and hypothesized that the rsFC of the SMA proper and preSMA may demonstrate different reorganization patterns in chronic stroke.

## Materials and Methods

### Subjects

The experiment was approved by the Ethical Committee of Tianjin Medical University General Hospital, and written informed consent was obtained from each subject before the study. Inclusion criteria were as follows: first-onset stroke; single lesion of ischemic infarct involving the internal capsule and neighboring regions; manifested motor deficit at stroke onset; right-handed before stroke; time after stroke onset of >6 months; and well-recovered global motor function with upper extremity Fugl–Meyer test (UE_FMT) of >60 and whole extremity Fugl–Meyer test (WE_FMT) of >90. Exclusion criteria included recurrent stroke after first onset, with any other brain abnormalities, and a history of drug dependency or psychiatric disorders. According to these criteria, 25 patients (seven females and 18 males; mean age: 56.2 years; range: 42–72 years) were finally included in this study (Table 1). The lesion location has been described in detail in an earlier work (Li et al., 2013). Twenty-two right-handed, age-matched, healthy controls (11 females and 11 males; mean age: 57.2 years; range: 47–74 years) were also recruited as controls who reported no history of psychiatric or neurological disorders. Compared with the stroke patients, healthy controls did not show any significant differences in both age (two-sample *t*-test: *t* = −0.50, *p* = 0.62) and gender (chi-square test: *χ^2^* = 2.40, *p* = 0.12).

**Table 1:** Demographic information, clinical data, lesion information, and clinical scores.

ID	Gender	Age	Lesion	WE_FMT	Post medical history
001	F	65	Right IC, CR	94	HD, HL
002	M	62	Right IC	100	HD, HL
003	F	63	Right CR, IC, LN	98	HBP
004	F	52	Right CR, IC, LN	99	None
005	M	53	Right CR, IC, LN	95	HBP, HL
006	M	65	Right CR	98	HD, HL
007	M	59	Left CR, IC, LN	98	HD, HL
008	M	49	Left CR, IC, LN	98	HD, HD
009	M	60	Left CR	100	HD, HL
010	F	72	Right CR, IC, LN	98	HD
011	F	55	Left Th	100	HD
012	M	49	Right CR, IC, LN	100	HD
013	M	42	Left Th	100	HD, DM, TIA, HL
014	M	50	Left CR, IC	100	HD, TIA, HL
015	M	52	Left CR, IC	100	HL
016	M	58	Left IC	100	HBP
017	M	65	Right CR, IC, LN	99	HBP, HD
018	F	63	Right Th	100	HD
019	M	55	Left IC, LN	100	HBP, HL
020	M	47	Left CR	100	HBP, DM, HL
021	M	58	Right CR, Cau, IC	100	HD
022	M	63	Left CR, IC	100	TIA, HL, DM
023	M	45	Right CR	100	HD, DM, HL
024	M	49	Right CR, IC	96	HD, HL
025	F	53	Left CR, IC, LN	99	HBP

*Note: Cau, caudate; CR, corona radiata; DM, diabetes mellitus; HBP, high blood pressure; HD, hyperglycemia; HL, hyperlipidemia; IC, internal capsule; LN, lenticular nucleus; TIA, transient ischemic attack; Th, thalamus; WE_FMT, whole extremity Fugl–Meyer test*.

It should be noted that the dataset of the present study has been applied in an early study that aimed to elucidate the reorganization of the functional connectivity of the cognitive-related cerebellar subregions after chronic stroke (Li et al., 2013). Except for the overlapped dataset and similar connectivity-based method, the scientific questions, parcellation methods, major findings, and results interpretation in the present study were much different from those in the early study by Li et al. (2013).

### Data Acquisition

Sagittal three-dimensional (3-D) T1-weighted images were acquired by a brain volume (BRAVO) sequence with the following parameters: repetition time (TR)/echo time (TE) = 8.1/3.1 ms; field of view (FOV) = 256 × 256 mm^2^; matrix = 256 × 256; slice thickness = 1.0 mm, no gap; 176 slices. The resting-state fMRI data of all of the subjects were obtained using a gradient-echo single-shot echo-planar imaging sequence (GRE-SS-EPI) with the following parameters: TR/TE = 2,000/30 ms; slice thickness = 3 mm, 1 mm gap; matrix = 64 × 64; FOV = 240 × 240 mm^2^; 38 transverse slices; 180 volumes. During fMRI scans, all subjects were instructed to keep their eyes closed, to stay as motionless as possible, to think of nothing in particular, and to not fall asleep. Before the data preprocessing, we flipped the imaging data from left to right along the midsagittal line for patients with lesions on the left hemisphere. For all patients, the right side was the ipsilesional hemisphere, whereas the left side corresponded to the contralesional hemisphere.

#### Preprocessing of fMRI Data

The resting-state fMRI data were preprocessed using the Statistical Parametric Mapping (SPM8^1^) and Data Processing Assistant for Resting-State fMRI (DPARSF; Chao-Gan and Yu-Feng, 2010). The first 10 volumes from each subject were discarded to allow the signal to reach equilibrium and the participants to adapt to the scanning noise. The remaining 170 volumes were corrected for acquisition time delay between slices. Then, head motion parameters were estimated; none of the 47 subjects had a maximum displacement of >2 mm or a maximum rotation of >2.0°. We also calculated framewise displacement (FD), and there was no significant difference between the healthy and patient group on the FD value (*t* = −0.828, *p* = 0.412). A unified segmentation approach was used to spatially normalize these functional images. The approach included the following steps: individual structural images were coregistered to the mean functional image after motion correction; the transformed structural images were segmented into gray matter, white matter, and cerebrospinal fluid using a unified segmentation algorithm; and the functional volumes were spatially normalized to the Montreal Neurological Institute (MNI) space using the normalized parameters estimated during segmentation. The functional images were then resampled into a voxel size of 3 × 3 × 3 mm^3^ and were smoothed using a Gaussian kernel of 8 × 8 × 8 mm^3^ full width at half maximum. Several sources of spurious variance, including the estimated motion parameters, the linear drift, and the average blood oxygenation-level-dependent (BOLD) signals in the ventricular, white matter regions, and the whole brain were removed from the data through linear regression. Finally, temporal band-pass filtering (0.01–0.08 Hz) was performed on the time series of each voxel to reduce the effects of low-frequency drift and high-frequency noise.

### Parcellation of the SMA

The human SMA mask was defined by the automated anatomical labeling (AAL) atlas (Tzourio-Mazoyer et al., 2002). The mask included gray matter on the medial wall and extended from *y* = −22 to *y* = 30 and from a short distance above the cingulate sulcus to the dorsal surface of the brain (Johansen-Berg et al., 2004). To compare the altered functional connectivity of the SMA subregion precisely, we created an SMA atlas for the elderly group based on the following steps. Nine of the 22 healthy subjects were randomly selected for the SMA parcellation. Such a relatively small number of participants have previously been used for parcellation, yielding stable parcellation results (Klein et al., 2007). On each subject’s individual space, Pearson correlation coefficients between the time series of each SMA voxel and the time courses of all other voxels of the whole brain were computed and converted to *z*-values using Fisher’s r-to-z transformation to improve the normality (Liu et al., 2017). Cross-correlation between the rsFC patterns of all voxels in the seed mask was calculated and used for automatic parcellation (Johansen-Berg et al., 2004). The cross-correlation matrix was fed into a spectral clustering algorithm with edge-weighted centroidal Voronoi tessellations (Wang et al., 2012) for automatic clustering. The goal of clustering the cross-correlation matrix was to group together voxels of the seed region that share similar connection profiles with other voxels of the brain. The number of component clusters was two, chosen by the experimenter. Considering interindividual differences, we calculated the maximum probability map (MPM) to show the final results (Caspers et al., 2008). To do this, we transformed each individual parcellation result from individual space to the MNI template. The MPM was calculated in the MNI space by assigning each voxel to the subregion to which it was most likely to belong. To validate if the involved nine subjects would affect stability of the parcellation, we further resegmented all of the 22 healthy subjects, and the MPM and centroid of each SMA subregion highly resembled the prior ones (Supplementary Table S1 and Supplementary Figure S1).

### The rsFC Pattern of Each SMA Subregion

Each region of interest (ROI) of the SMA and preSMA subregions was defined as a sphere (radius = 6 mm) centered at the averaged MNI coordinate of the centroid of each subregion. To ensure that all voxels of each ROI were within the gray matter, we multiplied each SMA ROI by the gray matter mask. For each subject of the two groups, Pearson correlation coefficients between the mean time series of each ROI and that of each voxel of the whole brain were computed and converted to *z*-values. Then, individual *z*-values were entered into a random effect one-sample *t*-test in a voxel-wise manner to identify brain regions that showed significant correlations with the seed ROI. Multiple comparisons were corrected for the false discovery rate (FDR) with a threshold of *p* < 0.05 and a cluster size of >30 voxels. Two-sample *t-test* with age and sex as nuisance covariates was performed to identify the rsFC differences of SMA subregions between the patient and healthy groups (*p* < 0.01, AlphaSim corrected). To validate the result, we preprocessed the data using the DPARSF (Chao-Gan and Yu-Feng, 2010) by normalizing the fMRI data into a symmetric MNI template and performed the same voxel-wise FC calculation and statistical analyses described above; for details, please see the Supplementary Methods.

To validate if the findings are replicable in left-sided and right-sided stroke patients, we additionally conducted ROI-wise analysis based on unflipped dataset. Each ROI of identified brain areas that had changed rsFC was defined as a sphere (radius = 6 mm) centered at the peak MNI coordinate. Then, we extract the ROI signal based on the no-flipped data in patients with left-sided and right-sided lesions and calculate the rsFC between these ROIs and the SMA subregions. A general linear model with age and sex as covariates was used to analyze if the rsFCs of the SMA subregions are statistically different between the controls and the patients with left-sided and right-sided lesions, separately (*p* < 0.05, uncorrected). To validate if the radius of ROI seeds would influence the FC analysis, we further defined the ROIs with a radius of 8 mm and calculated the FC and performed statistics as mentioned above. Finally, partial correlation analysis was conducted to explore the potential correlation between the altered rsFC of the SMA subregions and FMT scores with age and sex as nuisance covariates (*P* < 0.05, uncorrected).

## Results

### The SMA Subregions

**Figure 1:**
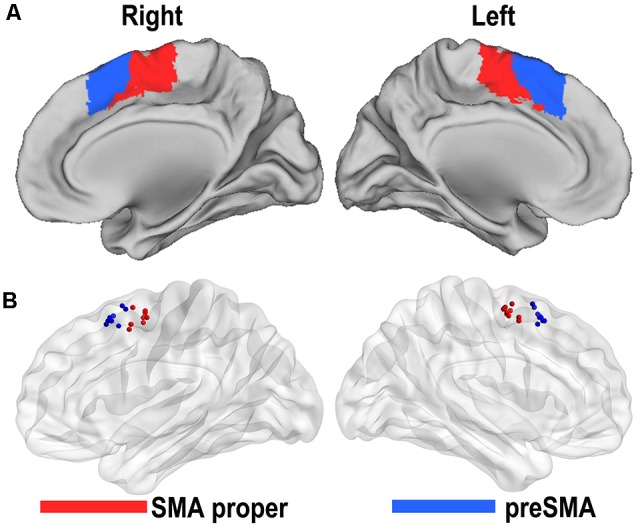
Functional connectivity-based parcellation of the human supplementary motor area (SMA). The human SMA can be subdivided into anterior (blue) and posterior (red) subregions, as shown in the maximum probabilistic maps of the right and left SMA **(A)**. Maps are displayed on a three-dimensional brain surface using the Caret software. Centroid distribution **(B)** of the SMA subregions. Maps are displayed on a three-dimensional brain surface using the Brainnet Viewer (Xia et al., 2013).

Based on the resting-state fMRI data and clustering algorithm, two separable subregions with different rsFC patterns along the anterior/posterior dimension were identified in the individual space for each subject. From the MPM of the SMA, we were able to identify an anterior subregion (preSMA) and a posterior subregion (SMA proper; Figure 1A). The centroid distribution of each SMA subregion across subjects is shown in Figure 1B. The averaged MNI coordinates of the centroids of the SMA proper and preSMA were as follows: the SMA proper (left: −4, −5, 59; right: 5, −5, 58) and the preSMA (left: −5, 16, 55; right: 4, 17, 55). The parcellations based on the 22 healthy subjects were shown in Supplementary Table S1 and Supplementary Figure S1, which highly resembled the one based on the prior parcellations. Thus, we still reported the result based on the prior ones.

### Whole Brain rsFC Pattern of the SMA Proper and PreSMA

**Figure 2:**
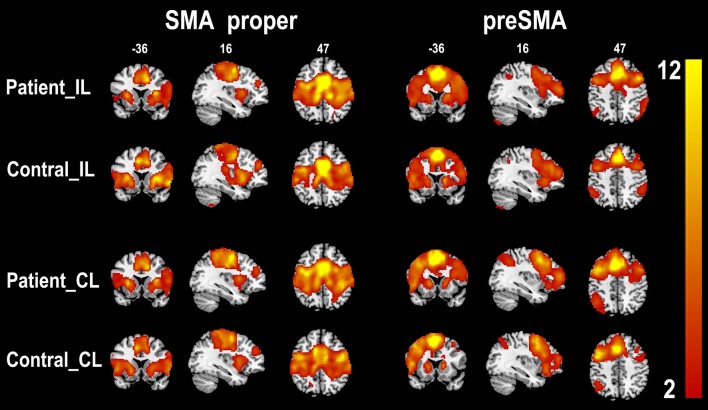
One-sample *t*-test shows the resting-state functional connectivity patterns of the SMA subregions (*p* < 0.05, FDR correction). CL, contralesional hemisphere; IL, ipsilesional hemisphere; SMA, supplementary motor area; FDR, false discovery rate.

The rsFC patterns of the bilateral SMA proper and preSMA in the two groups are shown in Figure 2. In healthy controls, the left and right SMA proper showed similar rsFC patterns. They showed rsFC with brain areas that belonged to the sensorimotor network (SMN), including the primary sensorimotor areas, cingulate motor areas (CMAs), caudate, putamen, thalamus, and pallidum, as well as brain areas that belonged to the salience network (SN), such as the fronto-insular cortex (FIC). In stroke patients, the SMA proper showed similar rsFC patterns as those in the healthy controls except that the patients showed weaker rsFC with the FICs than the healthy controls. The preSMA showed completely different rsFC patterns when compared to the SMA proper. In healthy controls, the preSMA was primarily correlated with three networks, including the frontoparietal (FP) network (such as the lateral PFC, and posterior parietal cortex), motor control network (such as the anterior CMA and basal ganglia), and the SN (such as the FICs). The rsFC patterns of the preSMA in stroke patients were also similar to those in healthy controls.

### Altered rsFC Patterns of SMA Subregions in Well-Recovered Stroke Patients

**Figure 3:**
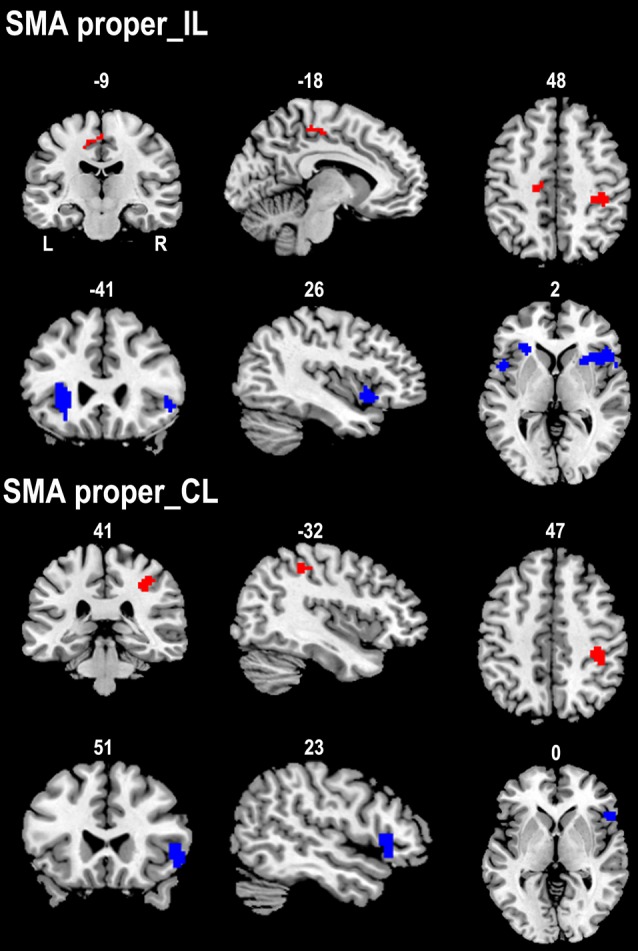
Contrast maps of the rsFCs of the SMA proper between stroke patients and healthy subjects. Red blobs and blue blobs indicate increased and decreased functional connectivity in patients compared with healthy subjects, respectively. CL, contralesional hemisphere; IL, ipsilesional hemisphere; rsFC, resting-state functional connectivity; SMA, supplementary motor area.

We compared the rsFC patterns of the SMA proper and preSMA between the two groups (Figure 3). The ipsilesional SMA proper exhibited increased rsFC with the ipsilesional primary sensorimotor area and the contralesional posterior CMA in stroke patients compared to healthy controls. Stroke patients also demonstrated decreased rsFC between the ipsilesional SMA proper and the bilateral FICs. Similarly, the contralesional SMA proper showed increased rsFC with the ipsilesional primary sensorimotor area and decreased rsFC with the ipsilesional FIC in stroke patients compared to healthy controls (Figure 3).

**Figure 4:**
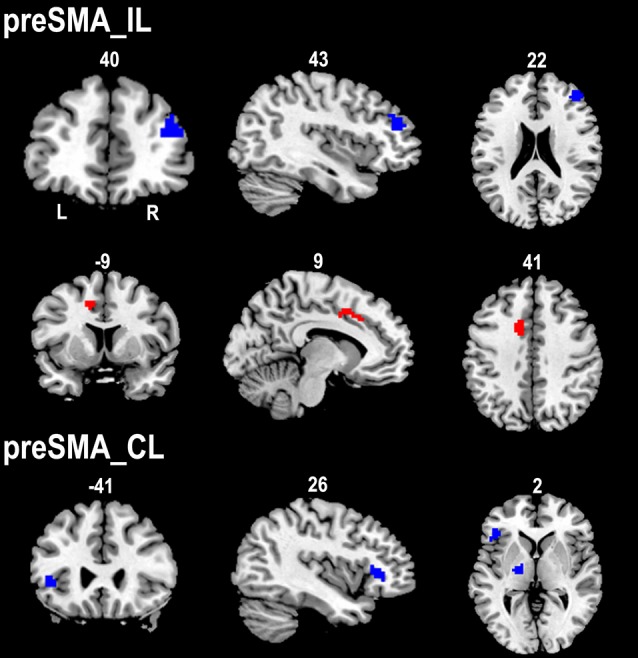
Contrast maps of the rsFCs of the preSMA between stroke patients and healthy subjects. Red blobs and blue blobs indicate increased and decreased functional connectivity in patients compared with healthy subjects, respectively. CL, contralesional hemisphere; IL, ipsilesional hemisphere; rsFC, resting-state functional connectivity; SMA, supplementary motor area.

The ipsilesional preSMA displayed increased rsFC with the contralesional anterior CMA and decreased rsFC with the ipsilesional dorsolateral prefrontal cortex (DLPFC) in stroke patients compared to healthy controls. However, stroke patients only showed decreased rsFC between the contralesional preSMA and contralesional thalamus and FIC when compared to healthy controls (Figure 4).

All of the above results were shown in Supplementary Table S2. To validate that the result would be influenced by the normalization method, we preprocessed the data using the DPARSF by normalizing the functional magnetic resonance imaging (fMRI) data into a symmetric MNI template, as shown in Supplementary Figure S2 and Supplementary Results, and the findings are consistent with the original ones to some extent. We additionally conducted an analysis based on unflipped dataset with different radii (6 vs. 8 mm) of ROI definition to validate if the initial results are replicable in patients with either left-sided lesions or right-sided lesions. As shown in Supplementary Figures S3, S4, the statistical significances based on the two different ROI definitions were much similar, suggesting that the radius of ROI definition may not affect the findings in the present study.

Partial correlation analysis did not find any correlation between the altered rsFC of SMA subregions and FMT scores (*P* > 0.05).

## Discussion

This is the first study to investigate rsFC changes of the SMA after stroke at the level of the subregion. In stroke patients, the SMA proper showed increased rsFCs with brain regions of the motor execution network, whereas the preSMA showed increased rsFC with the motor control network. These findings suggest that the two SMA subregions exhibit completely different functional reorganization patterns within the motor network. Additionally, we found that both the SMA proper and the preSMA showed decreased rsFC with brain areas involved in cognitive control, which may relate to the impaired cognitive function in stroke patients.

Consistent with previous studies (Johansen-Berg et al., 2004; Kim et al., 2010; Zhang et al., 2011), we have parcellated the SMA into anterior (preSMA) and posterior (SMA proper) clusters based on the rsFC patterns. The SMA proper functionally connected with brain areas that belonged to the motor execution network, which supports its function in motor execution (Maier et al., 2002; Krieghoff et al., 2009; Chouinard and Paus, 2010; Kim et al., 2010; Zhang et al., 2011). The preSMA functionally connected with the frontal, parietal, and insular areas that are closely related to cognitive control, including the control of complex motor behaviors (Kim et al., 2010; Zhang et al., 2011). The significant rsFC between the SMA proper and the bilateral FICs suggests that the SMA proper is also involved in motor control.

The increased activation in the SMA was found to contribute to motor recovery after stroke (Chollet et al., 1991; Jaillard et al., 2005). This hypothesis is validated by the findings that the initially decreased effective connectivity of the SMA (Grefkes et al., 2008; Rehme et al., 2011) was finally increased at the chronic stage of stroke (Mintzopoulos et al., 2009; Rehme et al., 2011; Zhang et al., 2016; Chen et al., 2018). The relationship between the rsFCs of the SMA and motor recovery in stroke patients is further validated in a longitudinal study. This study revealed that the rsFCs between the ipsilesional primary motor cortex and the contralesional SMA at onset was positively correlated with motor recovery at 6 months after stroke (Park et al., 2011). Although previous studies have revealed the functional connectivity changes of the SMA after stroke (Grefkes et al., 2008; Mintzopoulos et al., 2009; Park et al., 2011; Rehme et al., 2011), there is no study that focused on the rsFC changes of the SMA at the level of the subregion. In the present study, we revealed different patterns in increased rsFCs between the SMA subregions. The SMA proper showed increased rsFC with both the primary and secondary motor areas, whereas the preSMA only exhibited increased rsFC with the secondary motor areas. The discrepancy may be a reflection of the differences in the rsFC patterns and functions of these two SMA subregions.

As discussed above, the SMA subregions, especially the preSMA, are implicated in a motor control network that controls more complex motor behaviors. During the performance of a complex motor task, the sensory systems identify information about the performance (including errors and conflicts) and environment. The information from different sensory modalities is converged to the FICs to direct attention to pertinent stimuli as changing conditions, internal or external homeostatic demands, and context (Seeley et al., 2007); and then the FICs (especially the right FIC) initiate the activity of the brain regions associated with cognitive control (Seeley et al., 2007). These brain areas typically include the anterior cingulate cortex, SMA (especially the preSMA), and prefrontal areas where the errors are corrected and the conflicts are resolved. Finally, the correct control signals send to the motor control system. The process is recycled repeatedly to ensure the smooth execution of a complex motor task. Our findings of decreased rsFCs of the SMA subregions with the FICs and DLPFC in well-recovered stroke patients may suggest the dysfunction of the motor control network. The connectivity deficits within the cognitive control network may result in the deficits even in well-recovered stroke patients when they are performing complex motor tasks. This hypothesis is supported by the findings that well-recovered stroke patients also exhibited functional deficits in fine or complex motor tasks (Gerloff et al., 2006; Ustinova et al., 2006; Lotze et al., 2011; Verbraak et al., 2012). Our findings are consistent with previous rsFC studies that revealed decreased rsFC (Yin et al., 2012) or resting-state effective connectivity (Inman et al., 2012) of the motor-related areas with prefrontal and parietal brain areas associated with cognitive control. Besides, our research group have found the disconnection between the cerebellar subregion crus II with the cognitive control frontoparietal network, which may explain the deficits in cognitive control function (Li et al., 2013). Although our findings suggest rsFC deficits within the motor control network even in well-recovered stroke patients, future studies should be performed on the relationship between the rsFC deficit and the fine cognitive dysfunction in a more direct way.

### Limitations

There are several limitations that should be noted in the present study. First, this study has a cross-sectional design, and lack of the data (e.g., FMT scores and resting-state fMRI) from the patients in the (sub)acute stage, therefore, makes it difficult to draw conclusions about the relationship between the rsFC changes of the SMA subregions and motor recovery. Investigation of the dynamic changes of the SMA subregions after subcortical stroke should be performed in future studies. Second, there is a lack of cognitive assessments, which prevents us from investigating the direct relationships between the rsFC impairments within the cognitive control network. Further studies should be performed to determine these relationships. Finally, due to the small sample size of stroke subgroups (12 left-sided stroke, 13 right-sided stroke), we had flipped the brains with left lesions to the right side to increase the statistical power. To validate if the findings are replicable in left-sided and right-sided stroke patients, we additionally conducted ROI-wise analysis based on the unflipped dataset; although the direction of rsFC change was consistent between the two stroke subgroups, the significance level was much weaker. Thus, it still needs to be extensively clarified in the future with a larger sample size.

## Conclusion

This is the first study to investigate the rsFC changes of the SMA subregions in well-recovered stroke patients. We found different rsFC changes in the SMA proper and preSMA after stroke. Both SMA subregions showed increased rsFC with motor-related areas that may be associated with the recovery of motor function. In contrast, both subregions showed decreased rsFC with brain areas of the motor control network that may underlie the dysfunction in complex motor behaviors even in these well-recovered stroke patients.

## Data Availability Statement

All datasets generated for this study are included in the article/Supplementary Material.

## Ethics Statement

The experiment was approved by the Ethical Committee of Tianjin Medical University General Hospital and written informed consent was obtained from each subject before the study.

## Author Contributions

HL and WQ designed the experiment and wrote the protocol and the draft of manuscript text. WC, HL, and LX performed image processing and statistical analyses. WC and WL collected the magnetic resonance imaging (MRI) data.

## Conflict of Interest

The authors declare that the research was conducted in the absence of any commercial or financial relationships that could be construed as a potential conflict of interest.

## Footnotes

^1^http://www.fil.ion.ucl.ac.uk/spm

## References

[B1] Alexander G. E. DeLong M. R. Strick P. L. (1986). Parallel organization of functionally segregated circuits linking basal ganglia and cortex. Annu. Rev. Neurosci. 9, 357–381. 10.1146/annurev.ne.09.030186.002041 3085570

[B2] Boehler C. N. Appelbaum L. G. Krebs R. M. Hopf J. M. Woldorff M. G. (2010). Pinning down response inhibition in the brain—conjunction analyses of the stop-signal task. NeuroImage 52, 1621–1632. 10.1016/j.neuroimage.2010.04.276 20452445 PMC2910135

[B3] Caspers S. Eickhoff S. B. Geyer S. Scheperjans F. Mohlberg H. Zilles K.. (2008). The human inferior parietal lobule in stereotaxic space. Brain Struct. Funct. 212, 481–495. 10.1007/s00429-008-0195-z 18651173

[B58] Chao-Gan Y. Yu-Feng Z. (2010). DPARSF: a MATLAB toolbox for “Pipeline” data analysis of resting-state fMRI. Front. Syst. Neurosci. 4:13. 10.3389/fnsys.2010.00013 20577591 PMC2889691

[B4] Chen J. Liu M. Sun D. Jin Y. Wang T. Ren C. (2018). Effectiveness and neural mechanisms of home-based telerehabilitation in patients with stroke based on fMRI and DTI: a study protocol for a randomized controlled trial. Medicine 97:e9605. 10.1097/md.0000000000009605 29504985 PMC5779754

[B5] Choi J. Y. Lee K. H. Na D. L. Byun H. S. Lee S. J. Kim H.. (2007). Subcortical aphasia after striatocapsular infarction: quantitative analysis of brain perfusion SPECT using statistical parametric mapping and a statistical probabilistic anatomic map. J. Nucl. Med. 48, 194–200. 10.1146/annurev.fluid.34.081701.134821 17268014

[B6] Chollet F. DiPiero V. Wise R. J. Brooks D. J. Dolan R. J. Frackowiak R. S. (1991). The functional anatomy of motor recovery after stroke in humans: a study with positron emission tomography. Ann. Neurol. 29, 63–71. 10.1002/ana.410290112 1996881

[B7] Chouinard P. A. Paus T. (2010). What have we learned from “Perturbing” the human cortical motor system with transcranial magnetic stimulation? Front. Hum. Neurosci. 4:173. 10.3389/fnhum.2010.00173 21060721 PMC2972749

[B8] Cummine J. Hanif W. Dymouriak-Tymashov I. Anchuri K. Chiu S. Boliek C. A. (2017). The role of the supplementary motor region in overt reading: evidence for differential processing in SMA-proper and pre-SMA as a function of task demands. Brain Topogr. 30, 579–591. 10.1007/s10548-017-0553-3 28260167

[B9] Dancause N. (2006). Vicarious function of remote cortex following stroke: recent evidence from human and animal studies. Neuroscientist 12, 489–499. 10.1177/1073858406292782 17079515

[B10] de Boissezon X. Demonet J. F. Puel M. Marie N. Raboyeau G. Albucher J. F.. (2005). Subcortical aphasia: a longitudinal PET study. Stroke 36, 1467–1473. 10.1161/01.str.0000169947.08972.4f 15933252

[B11] Dubovik S. Pignat J. M. Ptak R. Aboulafia T. Allet L. Gillabert N.. (2012). The behavioral significance of coherent resting-state oscillations after stroke. NeuroImage 61, 249–257. 10.1016/j.neuroimage.2012.03.024 22440653

[B12] Dum R. P. Strick P. L. (1991). The origin of corticospinal projections from the premotor areas in the frontal lobe. J. Neurosci. 11, 667–689. 10.1523/jneurosci.11-03-00667.1991 1705965 PMC6575356

[B13] Dum R. P. Strick P. L. (2005). Frontal lobe inputs to the digit representations of the motor areas on the lateral surface of the hemisphere. J. Neurosci. 25, 1375–13786. 10.1523/jneurosci.3902-04.2005 15703391 PMC6726000

[B14] Duncan P. W. Lai S. M. Keighley J. (2000). Defining post-stroke recovery: implications for design and interpretation of drug trials. Neuropharmacology 39, 835–841. 10.1016/s0028-3908(00)00003-4 10699448

[B15] Fan L. Li H. Zhuo J. Zhang Y. Wang J. Chen L.. (2016). The human brainnetome atlas: a new brain atlas based on connectional architecture. Cereb. Cortex 26, 3508–3526. 10.1093/cercor/bhw157 27230218 PMC4961028

[B16] Galea M. P. Darian-Smith I. (1994). Multiple corticospinal neuron populations in the macaque monkey are specified by their unique cortical origins, spinal terminations and connections. Cereb. Cortex 4, 166–194. 10.1093/cercor/4.2.166 8038567

[B17] Gerloff C. Bushara K. Sailer A. Wassermann E. M. Chen R. Matsuoka T.. (2006). Multimodal imaging of brain reorganization in motor areas of the contralesional hemisphere of well recovered patients after capsular stroke. Brain 129, 791–808. 10.1093/brain/awh713 16364955

[B18] Gottesman R. F. Hillis A. E. (2010). Predictors and assessment of cognitive dysfunction resulting from ischaemic stroke. Lancet Neurol. 9, 895–905. 10.1016/s1474-4422(10)70164-2 20723846 PMC3592203

[B19] Grefkes C. Fink G. R. (2014). Connectivity-based approaches in stroke and recovery of function. Lancet Neurol. 13, 206–216. 10.1016/S1474-4422(13)70264-3 24457190

[B20] Grefkes C. Nowak D. A. Eickhoff S. B. Dafotakis M. Kust J. Karbe H.. (2008). Cortical connectivity after subcortical stroke assessed with functional magnetic resonance imaging. Ann. Neurol. 63, 236–246. 10.1002/ana.21228 17896791

[B21] He B. J. Snyder A. Z. Vincent J. L. Epstein A. Shulman G. L. Corbetta M. (2007). Breakdown of functional connectivity in frontoparietal networks underlies behavioral deficits in spatial neglect. Neuron 53, 905–918. 10.1016/j.neuron.2007.02.013 17359924

[B22] He S. Q. Dum R. P. Strick P. L. (1995). Topographic organization of corticospinal projections from the frontal lobe: motor areas on the medial surface of the hemisphere. J. Neurosci. 15, 3284–3306. 10.1523/jneurosci.15-05-03284.1995 7538558 PMC6578253

[B23] Inman C. S. James G. A. Hamann S. Rajendra J. K. Pagnoni G. Butler A. J. (2012). Altered resting-state effective connectivity of fronto-parietal motor control systems on the primary motor network following stroke. NeuroImage 59, 227–237. 10.1016/j.neuroimage.2011.07.083 21839174 PMC3195990

[B24] Jaillard A. Martin C. D. Garambois K. Lebas J. F. Hommel M. (2005). Vicarious function within the human primary motor cortex? A longitudinal fMRI stroke study. Brain 128, 1122–1138. 10.1093/brain/awh456 15728652

[B25] Johansen-Berg H. Behrens T. E. Robson M. D. Drobnjak I. Rushworth M. F. Brady J. M.. (2004). Changes in connectivity profiles define functionally distinct regions in human medial frontal cortex. Proc. Natl. Acad. Sci. U S A 101, 13335–13340. 10.1073/pnas.0403743101 15340158 PMC516567

[B26] Kim J. H. Lee J. M. Jo H. J. Kim S. H. Lee J. H. Kim S. T.. (2010). Defining functional SMA and pre-SMA subregions in human MFC using resting state fMRI: functional connectivity-based parcellation method. NeuroImage 49, 2375–2386. 10.1016/j.neuroimage.2009.10.016 19837176 PMC2819173

[B27] Klein J. C. Behrens T. E. Robson M. D. Mackay C. E. Higham D. J. Johansen-Berg H. (2007). Connectivity-based parcellation of human cortex using diffusion MRI: establishing reproducibility, validity and observer independence in BA 44/45 and SMA/pre-SMA. NeuroImage 34, 204–211. 10.1016/j.neuroimage.2006.08.022 17023184

[B28] Krieghoff V. Brass M. Prinz W. Waszak F. (2009). Dissociating what and when of intentional actions. Front. Hum. Neurosci. 3:3. 10.3389/neuro.09.003.2009 19277217 PMC2654019

[B29] Krüger D. Klapötke S. Bode S. Mattler U. (2013). Neural correlates of control operations in inverse priming with relevant and irrelevant masks. NeuroImage 64, 197–208. 10.1016/j.neuroimage.2012.09.018 22989624

[B30] Li W. Han T. Qin W. Zhang J. Liu H. Li Y.. (2013). Altered functional connectivity of cognitive-related cerebellar subregions in well-recovered stroke patients. Neural Plast. 2013:452439. 10.1155/2013/452439 23862075 PMC3703724

[B31] Liu F. Wang Y. Li M. Wang W. Li R. Zhang Z.. (2017). Dynamic functional network connectivity in idiopathic generalized epilepsy with generalized tonic-clonic seizure. Hum. Brain Mapp. 38, 957–973. 10.1002/hbm.23430 27726245 PMC6866949

[B32] Lotze M. Beutling W. Loibl M. Domin M. Platz T. Schminke U.. (2011). Contralesional motor cortex activation depends on ipsilesional corticospinal tract integrity in well-recovered subcortical stroke patients. Neurorehabil. Neural Repair 26, 594–603. 10.1177/1545968311427706 22140195

[B33] Lu M. T. Preston J. B. Strick P. L. (1994). Interconnections between the prefrontal cortex and the premotor areas in the frontal lobe. J. Comp. Neurol. 341, 375–392. 10.1002/cne.903410308 7515081

[B34] Luppino G. Matelli M. Camarda R. Rizzolatti G. (1993). Corticocortical connections of area F3 (SMA-proper) and area F6 (pre-SMA) in the macaque monkey. J. Comp. Neurol. 338, 114–140. 10.1002/cne.903380109 7507940

[B35] Maier M. A. Armand J. Kirkwood P. A. Yang H. W. Davis J. N. Lemon R. N. (2002). Differences in the corticospinal projection from primary motor cortex and supplementary motor area to macaque upper limb motoneurons: an anatomical and electrophysiological study. Cereb. Cortex 12, 281–296. 10.1093/cercor/12.3.281 11839602

[B36] Mintzopoulos D. Astrakas L. G. Khanicheh A. Konstas A. A. Singhal A. Moskowitz M. A.. (2009). Connectivity alterations assessed by combining fMRI and MR-compatible hand robots in chronic stroke. NeuroImage 47, T90–T97. 10.1016/j.neuroimage.2009.03.007 19286464 PMC2720432

[B37] Muakkassa K. F. Strick P. L. (1979). Frontal lobe inputs to primate motor cortex: evidence for four somatotopically organized “premotor” areas. Brain Res. 177, 176–182. 10.1016/0006-8993(79)90928-4 115545

[B38] Nachev P. Kennard C. Husain M. (2008). Functional role of the supplementary and pre-supplementary motor areas. Nat. Rev. Neurosci. 9, 856–869. 10.1038/nrn2478 18843271

[B39] Nakata H. Sakamoto K. Ferretti A. Gianni Perrucci M. Del Gratta C. Kakigi R.. (2008). Somato-motor inhibitory processing in humans: an event-related functional MRI study. NeuroImage 39, 1858–1866. 10.1016/j.neuroimage.2007.10.041 18083602

[B40] Obeso I. Wilkinson L. Teo J. T. Talelli P. Rothwell J. C. Jahanshahi M. (2017). Theta burst magnetic stimulation over the pre-supplementary motor area improves motor inhibition. Brain Stimul. 10, 944–951. 10.1016/j.brs.2017.05.008 28624346

[B41] Park C. H. Chang W. H. Ohn S. H. Kim S. T. Bang O. Y. Pascual-Leone A.. (2011). Longitudinal changes of resting-state functional connectivity during motor recovery after stroke. Stroke 42, 1357–1362. 10.1161/strokeaha.110.596155 21441147 PMC3589816

[B42] Rehme A. K. Fink G. R. von Cramon D. Y. Grefkes C. (2011). The role of the contralesional motor cortex for motor recovery in the early days after stroke assessed with longitudinal FMRI. Cereb. Cortex 21, 756–768. 10.1093/cercor/bhq140 20801897

[B43] Schaefer A. Kong R. Gordon E. M. Laumann T. O. Zuo X. N. Holmes A. J.. (2018). Local-global parcellation of the human cerebral cortex from intrinsic functional connectivity MRI. Cereb. Cortex 28, 3095–3114. 10.1093/cercor/bhx179 28981612 PMC6095216

[B44] Seeley W. W. Menon V. Schatzberg A. F. Keller J. Glover G. H. Kenna H.. (2007). Dissociable intrinsic connectivity networks for salience processing and executive control. J. Neurosci. 27, 2349–2356. 10.1523/jneurosci.5587-06.2007 17329432 PMC2680293

[B45] Sharma N. Baron J. C. Rowe J. B. (2009). Motor imagery after stroke: relating outcome to motor network connectivity. Ann. Neurol. 66, 604–616. 10.1002/ana.21810 19938103 PMC3791355

[B46] Stebbins G. T. Nyenhuis D. L. Wang C. Cox J. L. Freels S. Bangen K.. (2008). Gray matter atrophy in patients with ischemic stroke with cognitive impairment. Stroke 39, 785–793. 10.1161/strokeaha.107.507392 18258824

[B47] Tang Q. Li G. Liu T. Wang A. Feng S. Liao X.. (2015). Modulation of interhemispheric activation balance in motor-related areas of stroke patients with motor recovery: systematic review and meta-analysis of fMRI studies. Neurosci. Biobehav. Rev. 57, 392–400. 10.1016/j.neubiorev.2015.09.003 26344667

[B48] Tombari D. Loubinoux I. Pariente J. Gerdelat A. Albucher J. F. Tardy J.. (2004). A longitudinal fMRI study: in recovering and then in clinically stable sub-cortical stroke patients. NeuroImage 23, 827–839. 10.1016/j.neuroimage.2004.07.058 15528083

[B49] Tzourio-Mazoyer N. Landeau B. Papathanassiou D. Crivello F. Etard O. Delcroix N.. (2002). Automated anatomical labeling of activations in SPM using a macroscopic anatomical parcellation of the MNI MRI single-subject brain. NeuroImage 15, 273–289. 10.1006/nimg.2001.0978 11771995

[B50] Ustinova K. I. Fung J. Levin M. F. (2006). Disruption of bilateral temporal coordination during arm swinging in patients with hemiparesis. Exp. Brain Res. 169, 194–207. 10.1007/s00221-005-0136-5 16331509

[B51] Verbraak M. E. Hoeksma A. F. Lindeboom R. Kwa V. I. (2012). Subtle problems in activities of daily living after a transient ischemic attack or an apparently fully recovered non-disabling stroke. J. Stroke Cerebrovasc. Dis. 21, 124–130. 10.1016/j.jstrokecerebrovasdis.2010.05.012 21115361

[B52] Wang J. Becker B. Wang L. Li H. Zhao X. Jiang T. (2019). Corresponding anatomical and coactivation architecture of the human precuneus showing similar connectivity patterns with macaques. NeuroImage 200, 562–574. 10.1016/j.neuroimage.2019.07.001 31276799

[B53] Wang J. Fan L. Zhang Y. Liu Y. Jiang D. Yu C.. (2012). Tractography-based parcellation of the human left inferior parietal lobule. NeuroImage 63, 641–652. 10.1016/j.neuroimage.2012.07.045 22846658

[B54] Wang J. Yang Y. Fan L. Xu J. Li C. Liu Y.. (2015). Convergent functional architecture of the superior parietal lobule unraveled with multimodal neuroimaging approaches. Hum. Brain Mapp. 36, 238–257. 10.1002/hbm.22626 25181023 PMC4268275

[B55] Wang L. Yu C. Chen H. Qin W. He Y. Fan F.. (2010). Dynamic functional reorganization of the motor execution network after stroke. Brain 133, 1224–1238. 10.1093/brain/awq043 20354002

[B56] Wang Y. Isoda M. Matsuzaka Y. Shima K. Tanji J. (2005). Prefrontal cortical cells projecting to the supplementary eye field and presupplementary motor area in the monkey. Neurosci. Res. 53, 1–7. 10.1016/j.neures.2005.05.005 15992955

[B57] Xia M. Wang J. He Y. (2013). BrainNet viewer: a network visualization tool for human brain connectomics. PLoS One 8:e68910. 10.1371/journal.pone.0068910 23861951 PMC3701683

[B59] Yin D. Song F. Xu D. Peterson B. S. Sun L. Men W.. (2012). Patterns in cortical connectivity for determining outcomes in hand function after subcortical stroke. PLoS One 7:e52727. 10.1371/journal.pone.0052727 23285171 PMC3527607

[B60] Zhang S. Ide J. S. Li C. S. (2011). Resting-state functional connectivity of the medial superior frontal cortex. Cereb. Cortex 22, 99–111. 10.1093/cercor/bhr088 21572088 PMC3236794

[B61] Zhang Y. Liu H. Wang L. Yang J. Yan R. Zhang J.. (2016). Relationship between functional connectivity and motor function assessment in stroke patients with hemiplegia: a resting-state functional MRI study. Neuroradiology 58, 503–511. 10.1007/s00234-016-1646-5 26843179

