# Supplementary material for: Differential Reorganization of SMA Subregions After Stroke: A Subregional Level Resting-State Functional Connectivity Study
Source: Front Hum Neurosci. 2020 Feb 28;13:468. doi: 10.3389/fnhum.2019.00468 (PMC7059000; doi:10.3389/fnhum.2019.00468)
Supplement: Supplementary file 1 [file Data_Sheet_1.docx]

**Supplementary Materials**

**Supplementary Methods**

***Preprocessing of fMRI data with symmetric normalization***

Based on the flipped resting-state fMRI data, we used the Statistical Parametric Mapping (SPM8, http://www.ﬁl.ion.ucl.ac.uk/spm) and Data Processing Assistant for Resting-State fMRI (DPARSF) (Yan and Zang, 2010) to preprocess the data. After initial standard slice timing, realignment, and normalization steps (as shown in the method of the main text), then the data was further normalized to a symmetric MNI template according to the following steps: 1) get the T1 images in MNI space, 2) create a symmetric T1 template by averaging the mean T1 template (created in Step 1) with its flipped version, 3) normalize the T1 image in MNI space for each subject to the symmetric T1 template (created in Step 2), and apply the transformations to the functional data. The functional images were then smoothed using a Gaussian kernel of 8 × 8 × 8 mm^3^ full-width at half-maximum. Several sources of spurious variance, including the estimated motion parameters, the linear drift, and the average BOLD signals in the ventricular, white matter regions, and the whole brain, were removed from the data through linear regression. Finally, temporal band-pass filtering (0.01-0.08 Hz) was performed on the time series of each voxel to reduce the effects of low-frequency drift and high-frequency noise.

***The rsFC pattern of each SMA subregion***

Each region of interest (ROI) of the SMA and preSMA subregions was defined as a sphere (radius = 6 mm) centered at the averaged MNI coordinate of the centroid of each subregion. To ensure that all voxels of each ROI were within the gray matter, we multiplied each SMA ROI by the gray matter mask. For each subject of the two groups, Pearson correlation coefficients between the mean time series of each ROI and that of each voxel of the whole brain were computed and converted to z values. Then, individuals' z-values were entered into a random effect one sample t-test in a voxel-wise manner to identify brain regions that showed significant correlations with the seed ROI. Multiple comparisons were corrected for false discovery rate (FDR) with a threshold of p < 0.05 and a cluster size of > 30 voxels. Two sample t-test with age and sex as covariates was performed to identify the rsFC differences of SMA subregions between the patient and healthy groups (p < 0.01, AlphaSim corrected).

**Supplementary Results**

In order to validate the result would be influenced by the normalization method, we preprocessed the data using the DPARSF by normalizing the fMRI data into a symmetric MNI template and compared the rsFC patterns of the SMA proper and preSMA between the two groups. The ipsilesional SMA proper exhibited decreased rsFC between the ipsilesional SMA proper and the contralesional FICs. Similarly, the contralesional SMA proper also demonstrated decreased rsFC with the contralesional FIC in stroke patients compared to healthy controls. The ipsilesional preSMA displayed increased rsFC with the bilateral medial prefrontal cortex in stroke patients compared to healthy controls. Similarly, stroke patients showed increased rsFC between the contralesional preSMA and bilateral medial prefrontal cortex and anterior cingulate motor areas when compared to healthy controls (Supplementary Figure S2).

**Supplementary Tables**

Table S1. The MNI coordinates of the centroid of each SMA subregion.

| **Subregions** | **MNI Coordinates**  **(N = 9)** | | | **MNI Coordinates**  **(N=22)** | | |
| --- | --- | --- | --- | --- | --- | --- |
|  | **x** | **y** | **z** | **x** | **y** | **z** |
| R_SMA proper | 5 | -5 | 58 | 4 | -4 | 61 |
| L_SMA proper | -4 | -5 | 59 | -5 | -5 | 61 |
| R_preSMA | 4 | 17 | 55 | 5 | 18 | 53 |
| L_preSMA | -5 | 16 | 55 | -5 | 18 | 52 |

Table S2. Altered rsFC patterns of SMA subregions in in well-recovered stroke patients.

| **FC** | **Positive** | **MNI Coordinates (x, y, z)** | **t-value** | **Cluster size**  **(voxels)** | **Negative** | **MNI Coordinates**  **(x, y, z)** | **t-value** | **Cluster size**  **(voxels)** |
| --- | --- | --- | --- | --- | --- | --- | --- | --- |
| R_SMA proper | R_primary sensorimotor area | 33 -48 54 | 3.70 | 90 | R_FIC | 36 17 0 | -3.66 | 125 |
|  | L_ posterior CMA | -6 -20 54 | 4.25 | 76 | L_FIC | -45 10 0 | -3.26 | 102 |
| L_SMA proper | R_primary sensorimotor area | 33 -49 51 | 3.61 | 98 | R_FIC | 51 24 -3 | -3.71 | 89 |
| R_preSMA | L_ anterior CMA | -12 24 42 | 3.23 | 74 | R_ DLPFC | 39 39 30 | -3.91 | 105 |
| L_preSMA | - | - | - | - | L_Thalamus | -18 -12 0 | -3.56 | 69 |
|  | - | - | - | - | L_FIC | -48 21 6 | -4.42 | 75 |

Abbreviations: CMA, cingulate motor area; DLPFC, dorsolateral prefrontal cortex; FIC, fronto-insular cortex.

**Supplementary Figures**


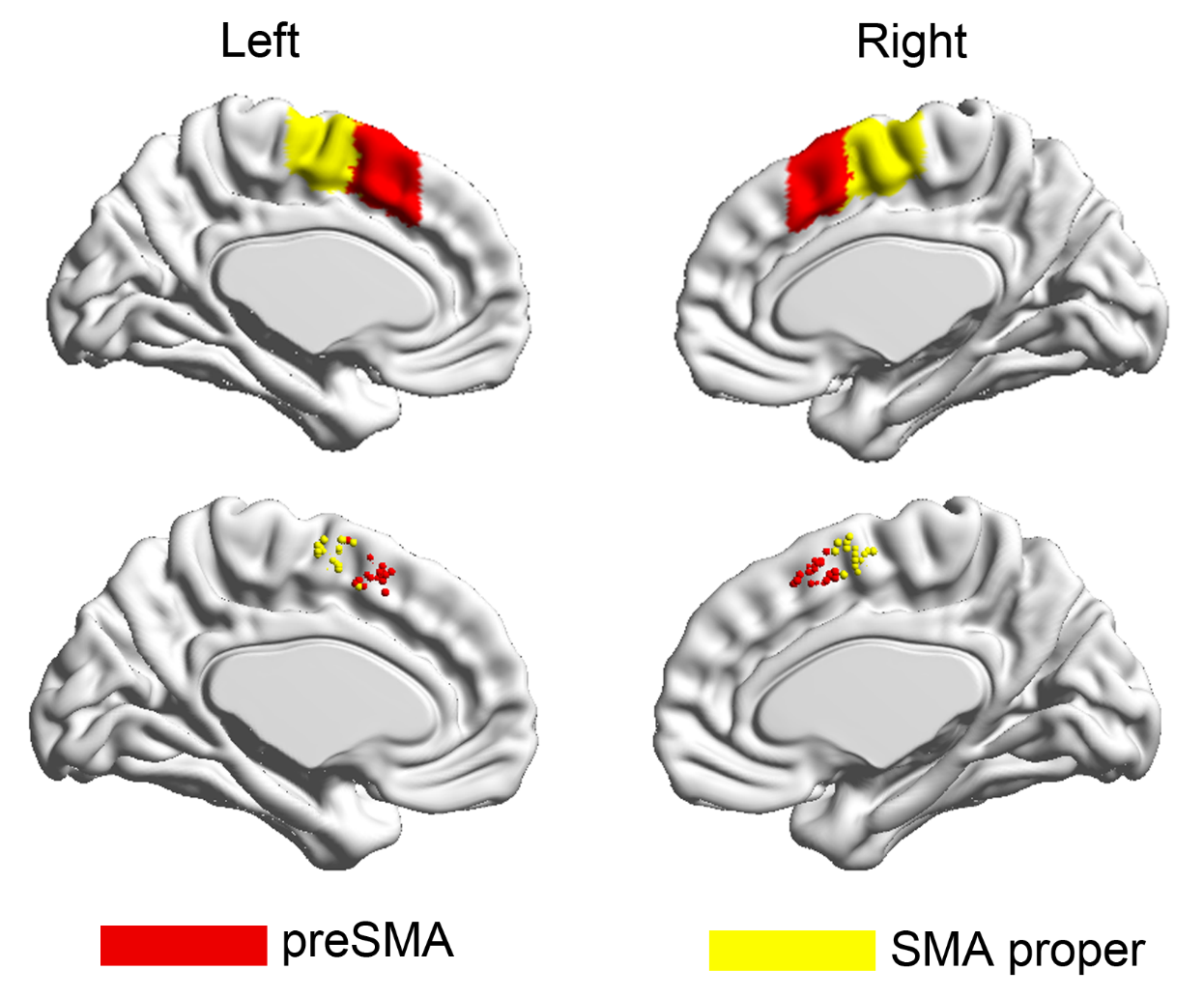


Figure S1. Functional connectivity-based parcellation of the human supplementary motor area (SMA) based on the 22 healthy subjects. The human SMA can be subdivided into anterior (red) and posterior (yellow) subregions, as shown in the maximum probabilistic maps of the right and left SMA. Centroid distribution of the SMA subregions. Maps are displayed on a three-dimensional brain surface using the Brainnet Viewer (Xia et al., 2013).


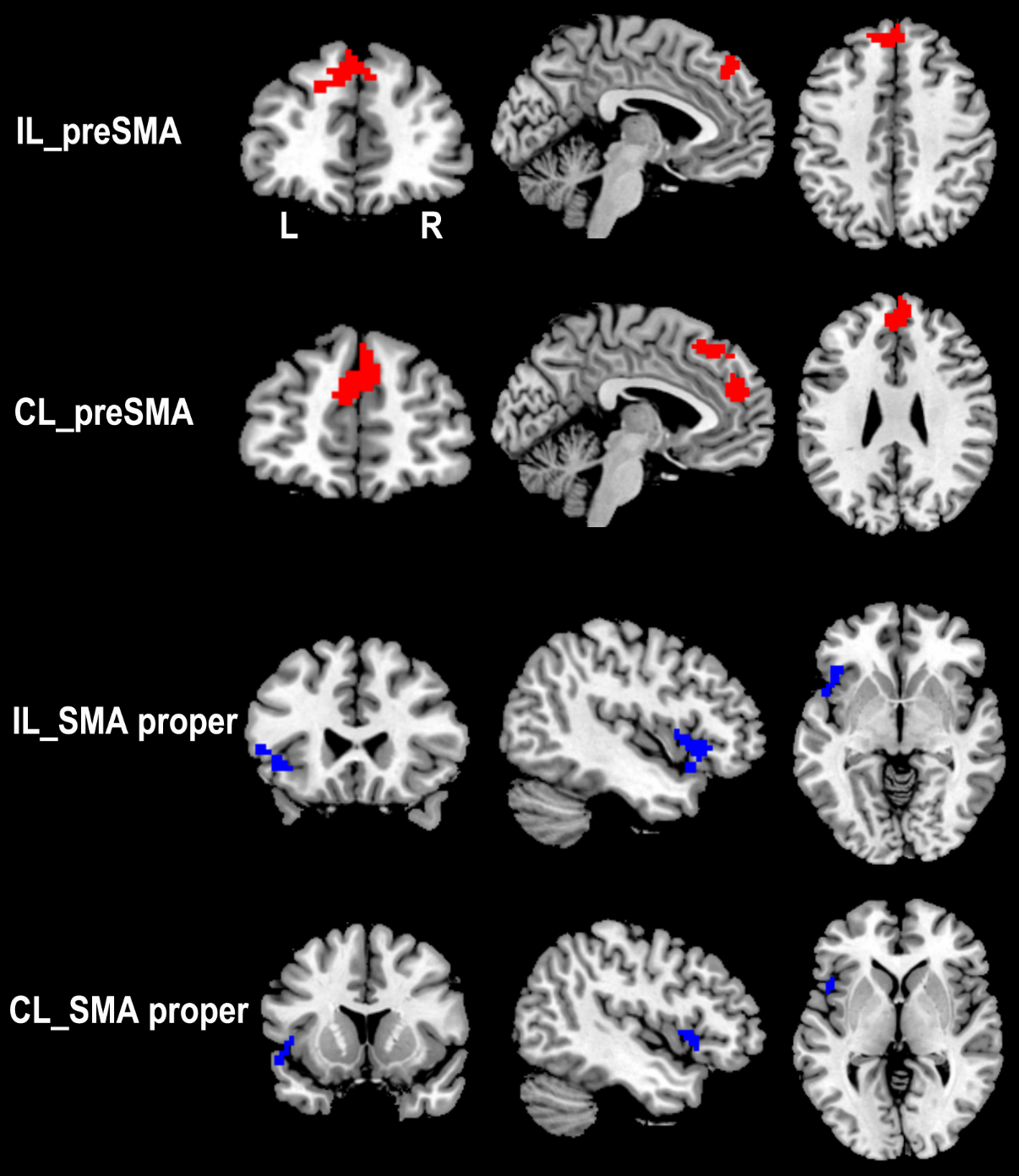


Figure S2. Contrast maps of the rsFCs of the preSMA and SMA proper between stroke patients and healthy subjects based on the data normalized to a symmetric MNI template. Red blobs and blue blobs indicate increased and decreased functional connectivity in patients compared with healthy subjects, respectively. CL, contralesional hemisphere; IL, ipsilesional hemisphere.


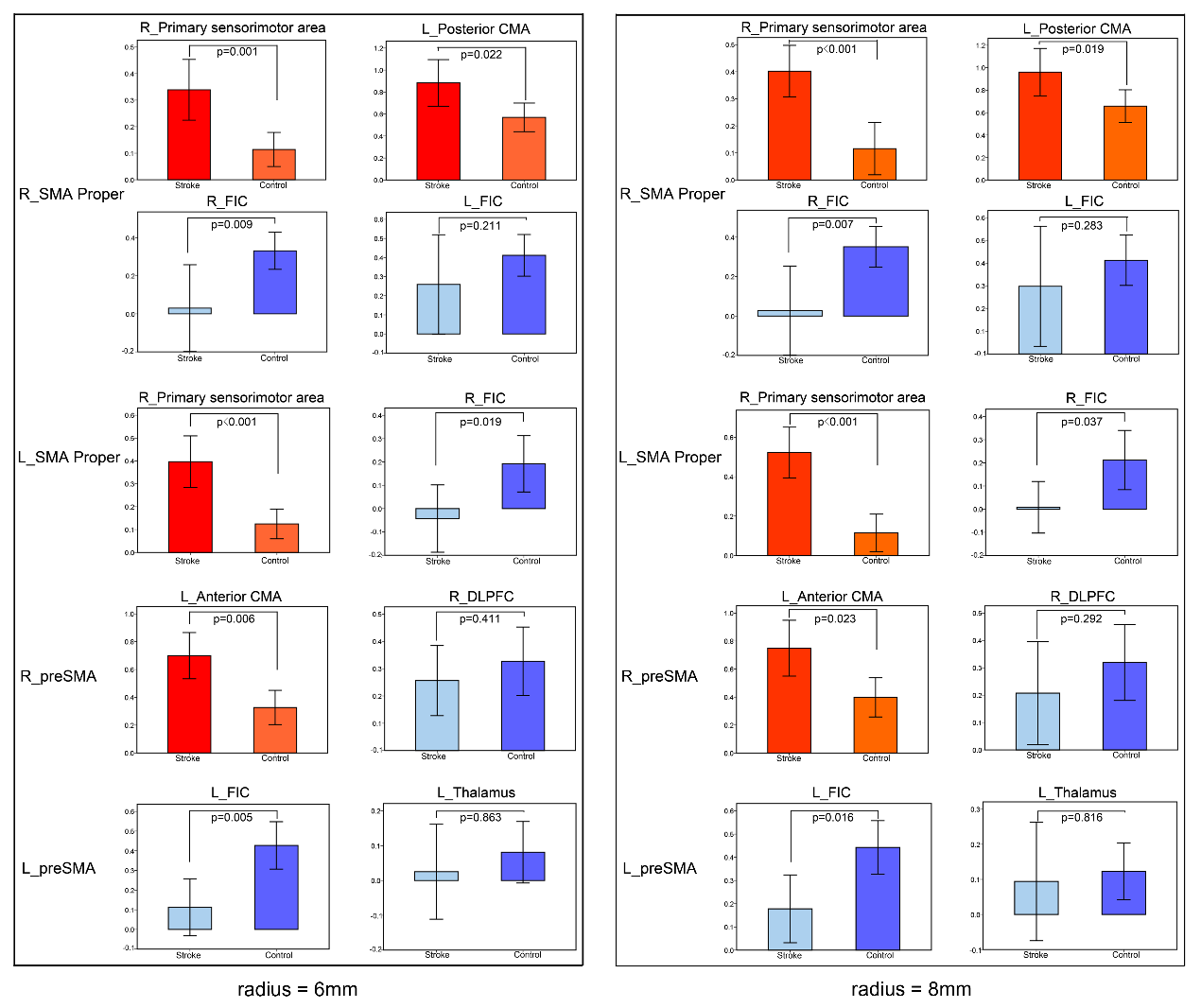


Figure S3. The validation based on the no-flipped data of the patients with left-sided lesions using SMA seed radius of 6 mm (Left panel) and 8 mm (Right panel).


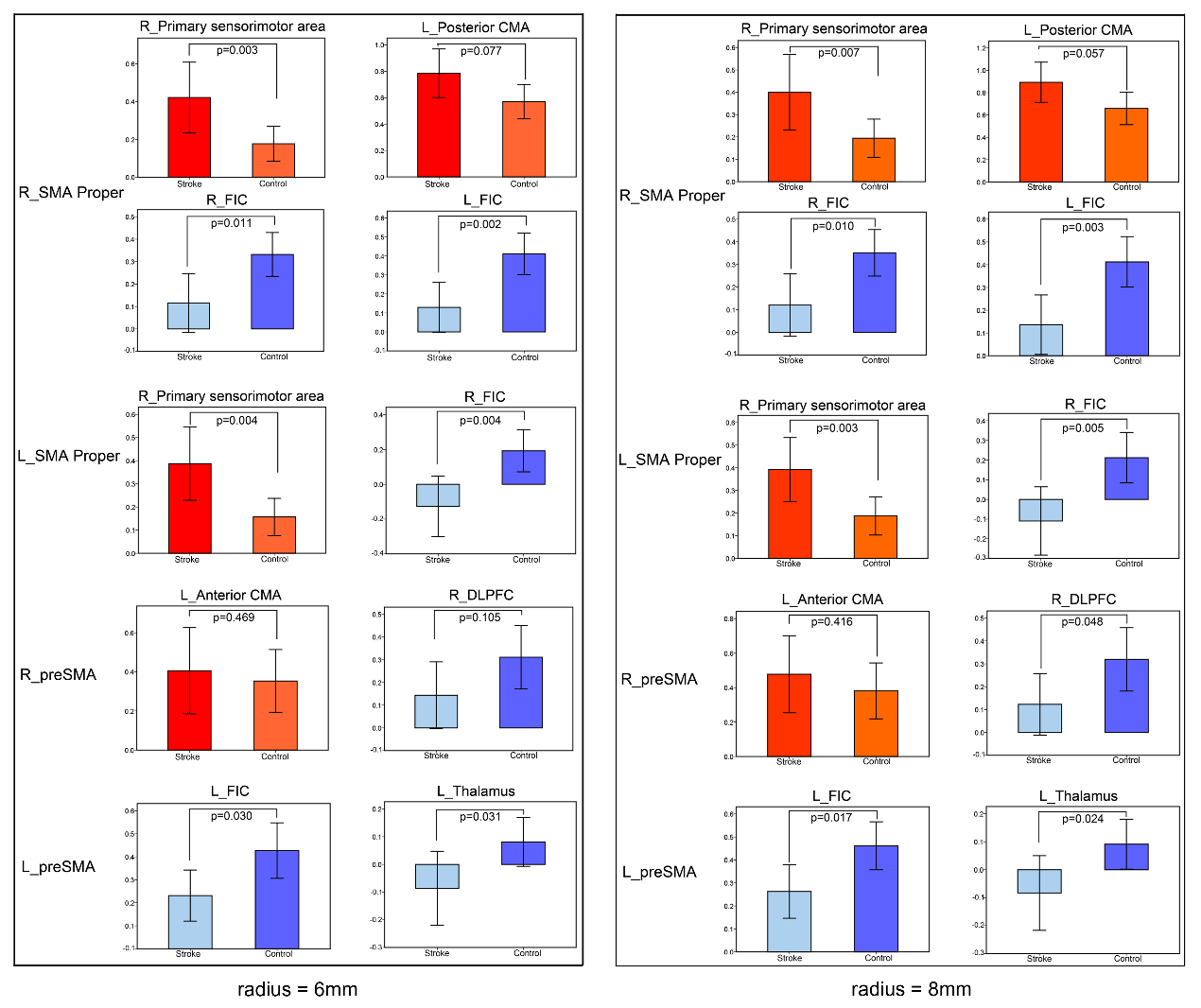


Figure S4. The validation based on the no-flipped data of the patients with right-sided lesions using SMA seed radius of 6 mm (Left panel) and 8 mm (Right panel).

**References**

Xia M, Wang J, He Y. (2013): BrainNet Viewer: a network visualization tool for human brain connectomics. *PLoS One* 8(7):e68910.

Yan C, Zang Y. (2010): DPARSF: A MATLAB Toolbox for "Pipeline" Data Analysis of Resting-State fMRI. *Front Syst Neurosci* 4:13.
